# A systematic review of the evidence that brain structure is related to muscle structure and their relationship to brain and muscle function in humans over the lifecourse

**DOI:** 10.1186/1471-2318-14-85

**Published:** 2014-07-10

**Authors:** Alixe HM Kilgour, Oliver M Todd, John M Starr

**Affiliations:** 1 Centre for Cognitive Ageing and Cognitive Epidemiology, University of Edinburgh, Room F2, 7 George Square, Edinburgh EH8 9JZ, UK; 2 Geriatric Medicine Unit, University of Edinburgh, Edinburgh, UK

## Abstract

**Background:**

An association between cognition and physical function has been shown to exist but the roles of muscle and brain structure in this relationship are not fully understood. A greater understanding of these relationships may lead to identification of the underlying mechanisms in this important area of research. This systematic review examines the evidence for whether: a) brain structure is related to muscle structure; b) brain structure is related to muscle function; and c) brain function is related to muscle structure in healthy children and adults.

**Methods:**

Medline, Embase, CINAHL and PsycINFO were searched on March 6th 2014. A grey literature search was performed using Google and Google Scholar. Hand searching through citations and references of relevant articles was also undertaken.

**Results:**

53 articles were included in the review; mean age of the subjects ranged from 8.8 to 85.5 years old. There is evidence of a positive association between both whole brain volume and white matter (WM) volume and muscle size. Total grey matter (GM) volume was not associated with muscle size but some areas of regional GM volume were associated with muscle size (right temporal pole and bilateral ventromedial prefrontal cortex). No evidence was found of a relationship between grip strength and whole brain volume however there was some evidence of a positive association with WM volume. Conversely, there is evidence that gait speed is positively associated with whole brain volume; this relationship may be driven by total WM volume or regional GM volumes, specifically the hippocampus. Markers of brain ageing, that is brain atrophy and greater accumulation of white matter hyperintensities (WMH), were associated with grip strength and gait speed. The location of WMH is important for gait speed; periventricular hyperintensities and brainstem WMH are associated with gait speed but subcortical WMH play less of a role. Cognitive function does not appear to be associated with muscle size.

**Conclusion:**

There is evidence that brain structure is associated with muscle structure and function. Future studies need to follow these interactions longitudinally to understand potential causal relationships.

## Background

Maintaining good levels of brain and muscle function across the lifespan is crucial to achieving a good quality of life [1-3]. There is substantial evidence showing an association between cognition and muscle function [4-8], however the role of muscle and brain structure within this association is less well understood. A greater understanding of this role will help to improve current knowledge of the mechanisms linking brain and muscle function over the lifecourse.

Several theories have been proposed as to why relationships between brain and muscle structure and function may exist. The common cause hypothesis postulates that there are core common underlying processes which drive ageing throughout the human body. The construct was originally described in a paper by Lindenberger and Baltes in 1994 who noted that measures of visual and auditory acuity accounted for variance in intelligence in old age [9]. Since then experiments in caloric restriction have demonstrated that the ageing process can be slowed down in multiple systems throughout the body by one intervention [10,11]. However, environmental factors also impact on how tissues change across the lifecourse and another theory by Mitnitski et al. proposes that the number of environmental stressors experienced (e.g. disease, smoking) and the ability to recover from them, vary the level of deficit accumulation experienced in multiple organ systems, and hence how tissues like brain and muscle change with age [12]. Potential underlying mechanisms include: pro-inflammatory cytokines (e.g. TNF-alpha and IL-6); the role of glucocorticoids and their intracellular amplifier 11beta-hydroxysteroid dehydrogenase type 1 [13-15]; the role of vitamin D [16,17]; exercise as a way to improve cardiovascular fitness in addition to its beneficial effect through hormones and cytokines [18-20]; and cellular senescence (e.g. through oxidative stress) [21,22].

In view of these theories, there should be a correlation between the structure and function of brain and muscle throughout our lifetime in the absence of significant pathology. This systematic review will search for studies that test the hypotheses that brain structure is related to muscle structure and/or function and that muscle structure is related to brain function in healthy children and adults. Previous studies and reviews have looked at evidence relating brain function (e.g. MMSE score) to muscle function (e.g. walking speed) therefore this separate but closely related field of literature will not be included in this review [5,23-25].

## Methods

The study protocol was published online in December 2011 at: http://www.ccace.ed.ac.uk/sites/default/files/Kilgouretal.pdf.

### Inclusion criteria

#### Population

All human subjects regardless of age were included in the study; from newborn babies to the oldest old, including post-mortem studies. This study is examining the relationship between brain and muscle in health, not within the effects of pathology therefore studies looking at how a disease affects brain or muscle were excluded. However studies which included a healthy control group, where the data from these subjects can be or was analysed separately were included. As morbidity increases in frequency with age it would be very restrictive to include solely those studies which include only participants who are free from any disease, therefore studies will be included provided the subjects have been recruited in a way that did not pre-dispose to morbidity being more prevalent than in the general population (e.g. from a diabetes clinic).

It was planned that subgroup analysis would be undertaken where possible and would include data being extracted to investigate the effects of gender, age, socioeconomic status and ethnicity.

#### Interventions/Comparators

Not applicable as the study is investigating normal physiology.

### Outcomes

#### Brain structure

•Whole brain volume (WBV) or total brain volume (TBV)

•Volume or cross sectional area of regions within the brain (e.g. hippocampus, frontal lobes)

•White matter integrity (e.g. White matter hyperintensities (WMH) or white matter signal abnormalities (WMSA)

•Histological findings about brain structure on autopsy

#### Brain function

•Any recognised measure of cognitive function including: memory, attention, executive function, language and processing speed

•Reaction time will not be used as this is dependent on aspects of brain and muscle structure and function

#### Muscle structure

•Muscle cross sectional area on CT, MRI or USS

•Muscle volume (using CT or MRI)

•Whole body lean tissue mass using DEXA, giving: total lean mass (TLM) or appendicular lean mass (ALM)

•Bioimpedance analysis (BIA)

•Histological findings on muscle biopsy or on autopsy

#### Muscle function

•Any recognised test of muscle strength, including isometric, isotonic, isokinetic tests

•Any recognised test of muscle power

•Functional tests of muscle function (e.g. usual or maximum gait speed)

### Study design

As this review is studying a physiological relationship, intervention studies were not included, unless they contained either a control arm with extractable data with no placebo treatment or baseline data prior to the intervention. Observational studies including cohort studies and cross sectional studies were included and the control arm of case control studies. Case reports were excluded as these would not contain evidence of normal physiological relationships out with pathology. The only other limiter used was “human” in Medline, Embase and PsycINFO but not Cinahl as it appears to screen out human studies erroneously.

### Search strategy

Database searches of Medline, Embase, CINAHL and PsycINFO were undertaken. All languages were included in the search. The Medline search strategy can be found in Appendix 1. The searches were all performed on 6th March 2014. A grey literature search was performed using Google and Google Scholar. Hand searching through citations and references of relevant articles was also undertaken.

### Study selection

The search was undertaken by two independent researchers. Titles +/− abstracts found using our search strategy were independently screened for relevance. The full text of the selected studies was reviewed against the inclusion criteria, and reasons for exclusion at this stage were recorded. At this point the two researchers met to discuss shortlists and discuss any articles which only one researcher had selected to decide if they should be included or not. Disagreements were resolved by consensus or adjudication by a third party (a Professor in Geriatric Medicine).

### Data extraction

The Clinical Fellow (AK) performed the data extraction using a data extraction sheet written by the Clinical Fellow and approved by the two co-authors (OT, JS).

### Contacting authors

Of the 84 studies found through our search, we wrote to 79 to request data or associations which were not given in the text. Five of the studies had given all the associations for the variables listed in the text. A letter was sent by email to either the corresponding author (after checking they were still working at the study location) or the last author (after the same checking process). Only one author was written to from each study (e.g. all articles arising from the Kansas Brain Aging Project, were grouped together when requesting extra data/associations). After the initial email a further email was sent around 2 weeks later to act as a reminder. Studies were given a minimum of around 1 month to reply.

Out of the 79 studies we wrote to: 25 studies (32%) sent either the requested data or associations; 22 (28%) replied stating they would try and send the data or associations to us but then never did; 12 studies (15%) replied stating they either no longer had access to the data or did not want to send either the requested data or associations to us; and 20 (25%) never replied to either of the emails.

### Quality assessment and risk of bias

All papers included in the study had their inclusion and exclusion criteria reviewed to check for possible bias in the study selection. The topic of the review is not looking at an intervention, therefore the risk of reporting bias for an individual paper is small. Also, in most of the papers, the relationship between muscle and brain was not the primary topic of the paper, further decreasing the risk of reporting bias. However when contacting the authors, asking for either the data or the associations, it was considered that the studies which replied may show some bias. The authors may look at their data and only reply if an association was found, or if they found a strong relationship they may not want this to be initially reported within a systematic review, but rather in a paper in its own right. All summary measures were included (e.g. odds ratio, beta).

### Data analysis

A narrative synthesis was completed. It was thought unlikely that the data would be comparable enough to allow meta-analysis (i.e. different measures of cognition, different muscle groups studied using different machines) and this proved correct. It was hoped that sub-group analysis would be undertaken, either in the form of a meta-analysis or more likely as a narrative synthesis for the reasons mentioned in the above paragraph.

## Results

**Figure 1:**
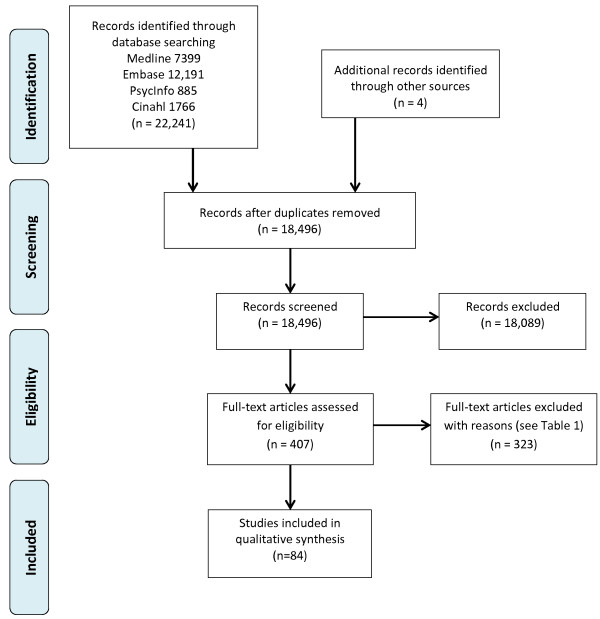
PRISMA flow diagram showing study selection.

The search results are presented in the PRISMA flow diagram in Figure 1. Reasons for exclusion of articles after reviewing the full text are reported in Table 1. After applying the inclusion and exclusion criteria 84 articles were identified; 53 articles either reported the appropriate associations or sent us the data or associations requested (Tables 2, 3 and 4), and 31 articles contained the required data but did not report the association between them and did not supply either the data or associations requested (Table 5). Out of the 53 articles which could be included in the review; 6 contained data on brain structure and muscle structure (Table 2); 33 contained data on brain structure and muscle function (Table 3); and 14 contained data on brain function and muscle structure (Table 4).

**Table 1:** Full-text articles excluded, with reasons

**Reason**	**Number**
Selected subjects (e.g. all had hip fracture, all had dementia etc.)	73
No measure brain or muscle structure	57
Review article, no relevant references	56
No measure muscle structure or function	47
Abstract, no published results within timeframe or irrelevant	34
No measure brain structure or function	19
Protocol paper, no published results within timeframe	12
Anthropometry only measure of structure	10
Letter or editorial, no results	7
Technique or theory paper	5
Case Report	2
No full text available	1
**Total**	**323**

**Table 2:** Studies identified with brain structure (+/− brain function) and muscle structure

**Authors**	**Year**	**Country and dataset**	**n**	**Study design**	**Mean age (sd)**	**Male (%)**	**Brain structure/Function**	**Muscle structure**	Associations*
**1. Heymsfield et al. **[26]	2012	Germany	260	Cross-sectional study	M 45.1 (14.9), F 38.6 (13.7)	43.1	Structure: Brain volume transformed into mass using 1.036 g/cm^3^	DEXA for FFM	Study: Linear regression models found that after adjusting for age and fat mass, FFM predicted brain mass in men (beta 0.023, R^2^ 5%, p = 0.01) and women (beta 0.003, R^2^ 6%, p = <0.0001).
							Function: not measured		
**2. Kilgour et al. **[27]	2013	UK, MacLullich Healthy Elderly Men Study	51	Longitudinal ageing study	73.8 (1.5)	100	Structure: Whole brain, hippocampal, ventricular, cerebellar volumes and ICV	Neck muscle CSA on MR head scan	Study: Total neck muscle CSA was found to predict 17% of the variance in whole brain volume (t = 2.86, p = 0.01). However, total neck muscle CSA did not significantly predict the variance in ventricular, hippocampal or cerebellar volumes (p > 0.05). Total neck muscle CSA did not significantly predict variance in either the memory factor or the cognitive processing factor (p > 0.05), however, it did predict 10% of the variance in the NART score (t = −2.12, p < 0.05). Adjusting for age, sex, ICV and NART where appropriate.
							Function: 9 tests of cognitive function reduced to 2 factors (cognitive processing factor and memory factor)		
**3. Wetmore et al. **[28]	2011	USA, Kansas, Brain Aging Project	60	2 year observational case–control study (Alzheimer’s dementia vs. non-dementia)	73.0 (7.2)	43.4	Structure: MRI for WM, GM, CSF, WBV and ICV	DEXA for lean mass and ASM (just arms and legs)	Study: none
							Function: Logical Memory I & II, Free & Cued Selective Reminding Task, Boston naming test, Verbal fluency, Digit span forward and backward, Letter-number sequencing, Trail making A & B, Stroop color-word test and Block design, MMSE		Calculated: Non-demented group only. WBV, GM volume and hippocampal volume not predicted by TLM adjusting for age, sex and ICV +/− education. WM volume was predicted by TLM (t 3.12, p = 0.003, partial eta squared 14%) adjusting for age, sex and ICV. TLM did not significantly predict global cognitive score or MMSE, adjusting for age and sex. Adjusting for height and education did not affect this.
**4. Burns et al. **[29]	2010	USA, Kansas, Brain Aging Project	70	Cross-sectional case–control study (Alzheimer’s dementia (AD) vs. non-dementia)	73.3 (7.3)	42.9	Structure: MRI for WM, GM, CSF, WBV and ICV	DEXA for total lean mass	Study: Positive relationship between WBV and TLM when control and AD subjects grouped together (beta = 0.20, p < 0.001), adjusting for ICV, age and sex. This appears to be driven by WM (beta 0.19, p < 0.001) rather than GM (beta 0.06, p = 0.27) States this persists in just the control group but doesn’t give any statistics for this. Positive relationship between MMSE and global cognitive score (composite of the cognitive tests) and lean mass when grouping AD and control subjects together. States that controlling for dementia status attenuates these results, but no specific statistics given. Calculated: See Wetmore et al. (2011) for Kansas Brain Aging Project data analysis.
							Function: Logical Memory I & II, Free & Cued Selective Reminding Task, Boston naming test, Verbal fluency, Digit span forward and backward, Letter-number sequencing, Trail making A & B, Stroop color-word test and Block design, MMSE		
**5. Honea et al. **[30]	2009	USA, Kansas, Brain Aging Project	56 healthy controls	Cross-sectional case–control study (Alzheimer’s dementia vs. non-dementia)	73.3 (6.2)	41.1	Structure: MRI for GM, WM, CSF, WBV, hippocampal and parahippocampal volumes	DEXA for total lean mass	Study: none
							Function: MMSE		Calculated: See Wetmore et al. (2011) for Kansas Brain Aging Project data analysis.
**6. Weise et al. **[31]	2013	USA, Phoenix	76	Cross-sectional study	32.1 (8.8)	31.6	Structure: MRI brain volumes (GM, WM, CSF, regional GMV)	DEXA, FFMI (FFM/height^2^)	Study: Fat-free mass index (FFMI) was negatively associated with GMV of the bilateral temporal lobes, ventromedial prefrontal cortex (vmPFC) (mainly subgenual portion of the ACC) and caudolateral orbitofrontal cortex and unilaterally with the left insular cortex (all p < 0.01). After adjusting for percentage body fat and fat mass, negative associations of FFM with GMV of the right temporal pole and bilateral vmPFC remained. All models adjusted for age, sex and handedness.
							Function: not measured		

*Associations column key: Study = results published within the study; Calculated = study authors supplied raw data to us and we performed the analysis.

**Table 3:** Studies identified with brain structure and muscle function

**Author**	**Year**	**Country and dataset**	**n**	**Study design**	**Mean age (sd)**	**Male (%)**	**Brain structure**^**#**^	**Muscle function**	Associations*
***Brain structure and grip strength***									
**1. Sachdev et al. **[32]	2009	Australia, PATH through life project	432	Observational cohort study	M 62.61 (1.42) F 62.62 (1.44)	52.8	Volumes of GM, WM and CSF, ICV and TBV (GM plus WM). Brain atrophy and subcortical atrophy, WMH	Grip strength in writing hand	Study: Total brain WMH volume predicted grip strength in men (beta −0.140, delta R^2^ 0.019, p < 0.05) but not in women (beta −0.140, delta R^2^ 0.018, p > 0.05).
**2. Anstey et al. **[33]	2007	Australia, PATH through life project	432	Observational cohort study	62.63 (1.43)	51.6	Total, anterior, midbody and posterior corpus callosum (CC) area	Grip strength in writing hand	Study: Grip strength adjusted for sex and ICV was found to correlate with CC midbody area (r = 0.103, p < 0.05), however CC total area and anterior and posterior CC areas did not significantly correlate with grip strength (p > 0.05).
**3. Sachdev et al. **[34]	2006	Australia, PATH through life project	469	Observational cohort study	M 62.56 (1.44) F 62.53 (1.47)	51.8	Volumes of GM, WM and CSF, ICV and TBV (GM plus WM). Brain atrophy and subcortical atrophy, WMH	Grip strength in writing hand	Study: None, see other articles from the PATH through life project for analysis using this dataset.
**4. Sachdev et al. **[35]	2005	Australia, PATH through life project	478	Observational cohort study	M 62.56 (1.44) F 62.54 (1.47)	52.3	WMH, ICV	Grip strength in writing hand	Study: Total brain WMH significantly predicted grip strength (beta −0.09, p = 0.002) adjusted for age, sex and depression. Correcting for comorbidity, cognition and brain atrophy did not attenuate the results (beta −0.13, p =0.001).
**5. Doi et al. **[36]	2012	Japan	110	Cross-sectional study	75.4 (7.1)	50	GM, WM, CSF, brain atrophy (measured using healthy volunteers)	Grip strength	Study: A MLR model found that grip strength is not related to brain atrophy (beta −0.082 (SE 0.005) p = 0.54). Adjusting for age, gender, BMI, education, MMSE, Tokyo Metropolitan Institute of Gerontology Index of Competence, geriatric depression scale and change in walking whilst dual tasking. No other associations given.
**6. Hardan et al. **[37]	2003	USA, Philadelphia	41 controls	Case–control study	18.6 (8.6)	Not given	Caudate, putamen and total brain volume	Grip strength	Study: Non-significant trends showed a negative correlation between right grip strength and total caudate volume (r = −0.303, p = 0.05) and left grip strength (r = −0.28, p = 0.07) in the control group. Not corrected for age or sex. No relationships given for other measures.
***Brain structure and gait speed***									
**7. Piguet et al. **[38]	2006	Australia, Sydney Older Person's Study	111	Longitudinal observational cohort study	M 85.29 (2.89) F 85.72 (3.41)	54.5	Cerebellar vermis area, (V1, V2 and V3 and total), Cerebellar volume, cerebral volume and ICV	Timed walk over 5 m, adjusted for lower limb arthritis	Study: None of the brain size measures (cerebellar vermis area, cerebellar volume or cerebral volume) significantly predicted timed walk (p > 0.05) after adjustment for age (but not sex, as was not deemed to be a significant contributor after univariate analyses).
**8. Callisaya et al. **[39]	2013	Australia, Tasmanian Study of Cognition and Gait (TASCOG)	225	Longitudinal cohort study	71.4 (6.8)	56.4	ICV, GM, WM-lesion free, hippocampal volume, WML	4.6 metre GaitRite computerized walkway (preferred speed)	Study: MLR were performed to investigate the relationship of longitudinal change in brain volumes and gait speed. They found that white matter atrophy (beta 0.25 (CI 0.09-0.40) p = 0.001), greater WML progression (beta −0.89 (CI −1.75- -0.02) p = 0.045), grey matter atrophy (beta 0.25 (CI 0.00-0.19) p = 0.06) and hippocampal atrophy (beta 0.01 (CI 0.00-0.02) p = 0.006) were all associated with a greater decline in gait speed.
**9. Srikanth et al. **[40]	2010	Australia, TASCOG	385	Longitudinal cohort study	72.2 (7.1)	56	WMLV, TBV	Gait speed using 4.2 m GAITRite system	Study: none, see Callisaya et al. (2013) for analysis using the TASCOG dataset.
**10. Srikanth et al. **[41]	2009	Australia, TASCOG	294	Longitudinal cohort study	72.3(7.0)	55.4	WMLV, TBV	Gait speed using 4.2 m GAITRite system	Study: none, see Callisaya et al. (2013) for analysis using the TASCOG dataset.
**11. Elbaz et al. **[42]	2013	France, Three-city study	4010	Cohort study	73.4 (4.6)	38.4	WML volumes	6 metre walk speed (usual and maximum)	Study: Logistic regression stratified by education found that high WML volumes were not associated with slow walking speed among highly educated participants (OR = 0.72), but were associated with a 2-fold-increased risk of slow walking speed among those with low education (OR = 3.19/1.61 = 1.99) (p interaction = 0.026), adjusted for sex, age and total WM volume. Results remained unchanged after adjustment for height, BMI, and MMSE score.
									Given: WM volume did not predict walking speed at baseline, adjusted for age, gender and ICV in a MLR (p > 0.05, n = 1510), or decline in walking speed over 7 years, adjusted for age, gender, ICV and baseline walking speed, (p > 0.05, n = 928). A logistic regression found that WM volume was not significantly associated with an increased risk of being in the quartile with the highest walking speed decline (p > 0.05).
**12. Dumurgier et al. **[43]	2012	France, Three-city study	1623	Cohort study	73.3 (4.1)	39.5	Regional grey matter volumes (sensorimotor cortex; frontal, parietal, temporal, occipital, and limbic lobes; insula; cerebellum; thalamus; basal ganglia nuclei, including the caudate nucleus, putamen and pallidum) and WMLs	Maximum walking speed over 6 metres	Study: A linear regression found that only basal ganglia volume (beta 0.075 (SE 0.025) p = 0.003) was significantly associated with walking speed; driven by caudate nucleus volume (beta 0.114 (SE 0.024) p < 0.001). All other regional GM volumes were not significantly associated with walking speed. A semi-bayes model found again only the basal ganglia volume (beta 0.061 (SE 0.028) p = 0.03) was significantly associated with walking speed; driven by caudate nucleus volume (beta 0.050 (se 0.019) p = 0.007). There was found to be a linear relationship between quartiles of caudate nucleus volume and faster walking speed (p for linear trend (0.001). These relationships were attenuated slightly for total basal ganglia volume by adjusting for MMSE and comorbidity plus smoking but not for caudate nucleus volume. All models adjusted for; age, sex, BMI, education level, ICV, volume of WMLs and silent infarcts. Given: See Elbaz et al. (2013) for Three-City Study data analysis.
**13. Dumurgier et al. **[44]	2010	France, Three-city study	Baseline 3604, f/u at 4y 1774	Cohort study	Baseline 73.4 (4.6) f/u 71.5 (3.6)	Baseline 38.1%, f/u 38.4%	WMH volume	Maximum walking speed over 6 metres, 1st and 4th follow up, mean 7 years	Study: none
									Given: See Elbaz et al. (2013) for Three-City Study data analysis.
**14. Soumare et al. **[45]	2009	France, Three-city study	1702	Cohort study	72.4 (4.1)	39.4	PVH, deep WMH and total WMH and total WM and ICV	Maximum walking speed over 6 metres, 1st and 4th follow up, mean 7 years	Study: A significantly lower mean walking speed was found in those with a total WMH volume above the 75th percentile compared to those below the 25th (Beta −0.026, p = 0.0003). A similar relationship was found for both deep WMH and PVH. A WMH volume greater than the 90th percentile more than doubled the risk of decline in walking speed compared with subjects with lower volumes of WMH (OR 2.6 (1.5-4.5), p = 0.001). This finding was replicated when looking at PVH but not for deep WMH volume. Given: See Elbaz et al. (2013) for Three-City Study data analysis.
**15. Starr et al. **[46]	2003	UK, ABC1921 cohort study	97	Longitudinal cohort study	78-79years	59.8	WMH in deep/subcortical, PVH and brain stem, Fazekas score	Self-paced time to walk 6metres	Study: A slower 6metre walk test was associated with increased brain stem lesions (F 7.11, p = 0.009, partial eta2 0.070), but not with WMH (deep) (F 3.33, p = 0.071) or PVH (F 2.47, p = 0.12). Doesn’t state if age and sex are adjusted for in these models. If HADS score and Raven’s score are adjusted for, brainstem lesions are no longer significantly associated with walking time.
**16. Manor et al. **[47]	2012	USA, Boston,	89 in control group	Case–control study	65.3 (8.2)	48.3	GM, WM, CSF, regional GM volumes; precentral and postcentral gyri, basal ganglia, cerebellum, and dorsolateral prefrontal cortex	75 metre walk test at preferred pace	Study: Within linear regression models, global GM volume and all of the regional GM volumes were not associated with walking speed in the control group (p > 0.005, Bonferroni adjusted). Adjusted for age, sex and body mass.
**17. Hajjar et al. **[48]	2010	USA, Boston, BP in stroke study (?overlap with Novak et al.)	Non-stroke group 43	Case–control observational study	68 (1)	44	WM, GM (global and regional), CSF normalized for ICV	Gait speed over 12mins at usual pace	Study: Gait speed was not significantly associated with GM volume (p = 0.85), but was significantly associated with WM volume (B = 1.30, p = 0.03) adjusting for age, gender, BMI and antihypertensive use.
**18. Novak et al. **[49]	2009	USA, Boston (?overlap with Hajjar et al.)	76	Observational study	64.7 (7.2)	47.4	GM, WM, CSF, WMH all as % brain tissue volume. WMH using Wahlund scale	Gait speed over 12mins at normal walking pace	Study: Gait speed was significantly associated with frontal WM normalized for brain tissue volume (R = 0.4, p = .003). Gait speed was significantly associated with frontal GM normalized for brain tissue volume (R = 0.3, p = .01). Adjusted for age and BMI (but not gender). Doesn’t say about other regional brain volumes, ie temporal etc. WMH volumes and PVH and punctuate scores were not associated with gait speed (p > 0.05).
**19. Moscufo et al. **[50]	2012	USA, Boston, Moscufo study – 2 year f/u	77	Longitudinal cohort study	84 (3.9)	40	WMH volume as % of ICV and regional WMH burden expressed as % of ROI volume. At baseline and 2y f/u.	Gait speed over 2.5 metres, maximum velocity and usual walking speed At baseline and 2y f/u.	Study: Total WMH burden was significantly associated with usual walking speed at baseline but not at follow-up, and maximum walking speed was not associated with total WMH at baseline or follow up. At baseline, regional WMH burden in the splenium of corpus callosum and anterior and superior corona radiata, was significantly associated with both walking measures (p < 0.05) and in addition the body of the corpus callosum was also associated with usual walking speed (p < 0.05). At follow-up, WMH burden in the splenium was significantly associated with both walking measures (p < 0.05) and in the body with maximum walking speed. Change in WMH burden, either total or in any of the 7 regional areas, over 2 years was not associated with a decline in usual walking speed (p > 0.1).
									Given: WMH burden is significantly associated with lower gait speed after adjustment for age, sex and BMI (rho = −0.327, p = 0.0008). WM/ICV is not significantly associated with gait speed with or without adjustment (p > 0.05). GM/ICV is significantly associated with gait speed with adjustment for age, gender and BMI (rho = 0.232, p < 0.05). CSF/ICV is significantly associated with gait speed with adjustment for age, sex, BMI (rho = −0.285, p = 0.004).
**20. Moscufo et al. **[51]	2011	USA, Boston, Moscufo study - baseline	99	Cross-sectional observational study	83(4)	42.4	WM, GM, WMH and CSF volumes all corrected for ICC. Brain atrophy. Regional WMH burden expressed as % of ROI volume.	Gait speed over 2.5metres (done as part of SPPB)	Study: Total WMH burden (i.e. % of ICV) correlates with gait speed (rho = −0.288, p = 0.004). Also all 9x regional burden measurements correlate with gait speed score too except sup. longitudinal fasciculus. No adjustment.
									Given: See Moscufo et al. (2012) for analysis using this dataset.
**21. Wolfson et al. **[52]	2005	USA, Boston, WML and mobility	28 at baseline, 14 at follow up	Prospective longitudinal observational study	SPPB 11or12 mean 81(1.7), SPPB = <8 mean 84(3.4)	64.3	GM, WM, WMSA, CSF, ICCV volumes	Gait velocity over 8metres	Study: Slower baseline gait velocity predicted more WMSA at visit 1 (p < 0.05), but not change in WMSA volume between visit 1 and 2 (p < 0.07). Significant negative relationship of between-visit change in gait velocity to CSF volume (r = 0.733, p < 0.005) and a positive relationship of between-visit change in gait velocity to WM volume (r = 0.558, p < 0.05). Betas not given. Brain volumes normalized for ICCV according to image processing section.
**22. Guttmann et al. **[53]	2000	USA, Boston, WML and mobility	28 (12 with SPPB score >10 and 16 < 9)	Observational cross-sectional study	SPPB > 10 79(5) SPPB < 9 83(6)	42.9	WM, WMSA, GM, CSF (normalized for ICCV)	Gait velocity over 8metres	Study: Gait velocity was not significantly predicted by age nor WMSA volume (no figures given or p value) adjusted with and without MMSE score.
**23. Rosano et al. **[54]	2012	USA, Cardiovascular health study	214	Longitudinal observational study	72.3 (3.8)	35.5	Brain volumes (GM, WMH, Prefrontal area, WM, CSF)	Timed 15 ft walk at usual pace	Study: Prefrontal area volume significantly predicted time to walk in a stepwise forward model (beta −0.15, p = 0.02).
**24. Barnes et al. **[55]	2009	USA, Cardiovascular Health Cognition Study, nested within the CVS Health Study	3375	Prospective, population-based, longitudinal study	75 (no sd)	41	White matter disease and ventricular enlargement	Gait speed over 15 ft	Study: none, see Rosano (2012), Rosano (2006), Rosano (2005) and Longstreth (1996) for analysis using the Cardiovascular Health study dataset
**25. Rosano et al. **[56]	2006	USA, Cardiovascular health study	321	Longitudinal observational study mean f/u 4 years	78.3 (no sd)	39.3	WMAs, ventricular enlargement	Gait speed at usual pace over 4 metres using GaitMat II	Study: Gait speed was significantly correlated to total WMAs (r = −.18, p < 0.0001) and white matter lesions in the brainstem (r = −.18, p = 0.01). After adjusting for age, slower gait speed was still significantly associated with white matter grade (p = 0.02). Logistic regression found that those in the lowest two quartiles of gait speed (ie < 1.02 m/s) had double the likelihood of having WMH graded 3 or above (p = 0.03), after adjustment for age, race, gender, and prevalent clinical CVD. VE graded >4 was not found to be significantly predicted by gait speed, however VE graded > 5 was, independent of age, gender, race and presence of CVD (OR = 2.91 for 1st vs. 4th quartile, OR 3.82 for 2nd vs 4th quartile)
**26. Rosano et al. **[57]	2005	USA, Cardiovascular health study	2450	Longitudinal observational study mean f/u 4 years	74.4 (4.7)	43	WMH and ventricular enlargement (graded as minimal, moderate and severe)	Gait speed over 15 ft at usual pace, starting from standing still	Study: Grade of ventricular enlargement was associated with baseline gait speed and mean change in gait speed/year. Gait speed decline was 2.5x that for those with severe VE than minimal VE. (p < 0.001). Grade of WMH was associated with baseline gait speed and mean change in gait speed/year (p = 0.003). In both analyses adjustment had been made for age, sex, race and education and CV risk factors (BMI, systolic BP, antihypertensive meds, internal carotid wall thickness, and ETOH intake) and prevalent CV disease.
**27. Silbert et al. **[58]	2008	USA, Oregon Brain Aging Study	104	Longitudinal cross-sectional study	85.1 (5.6)	38.5	PV WMH and s/c WMH, total WMH, brain volume, CSF volume, hippocampal volume, ICV	Gait speed over 9 m. Self-selected pace.	Study: Adjusted for age and ICV, higher baseline total WMH vol. was associated with increased rate of change in timed walking in seconds (r^2^ = 0.08, p = 0.0052). This relationship became non-significant after adjustment for multiple comparisons to threshold p value. PVH volume is associated with increased rate of change in timed walk in seconds (r^2^ = 0.12, p = .0039). However, baseline subcortical WMH vol. was not related to change in gait performance over time. Higher rate of PVH accumulation is associated with increased rate of change of time to walk 9 m (r^2^ = 0.15, p = .0453). Adjusted for age, ICV and baseline WMH volume:
									Calculated: In an unadjusted GLM, gait speed was predicted by total brain, WMH and hippocampal volume (p < 0.001). The relationship remained significant after adjusting for sex, age, ICV and height, for total brain volume (t = 3.61, p = .004, partial eta squared 4.3%) and WMH (t = −2.80, p = 0.006, partial eta squared 4.4%) but not for hippocampal volume.
**28. Marquis et al. **[59]	2002	USA, Oregon Brain Aging Study	108	Longitudinal cross-sectional study	83.2 (7.9)	37	Total brain volume, hippocampal volume, ICV	Gait speed over 9 m. Self-selected pace.	Study: Negative correlation between hippocampal volume and time to walk 30 ft (r = −.12). No p value given.
									Calculated: See Silbert et al. (2008) for Oregon Brain Aging Study data analysis.
***Brain structure and gait speed plus grip strength or isometric knee extension strength (IKES)***									
**29. Rosano et al. **[60]	2010	Iceland, AGES-Reykjavik study	795	Longitudinal cohort study	M 75.6 (5.4) F 75.6 (5.7)	41.1	MTR, ICV, brain parenchyma volume, semiquantitative subcortical WMH and PVH and total WMH volume, brain atrophy index	Gait speed over 6 m usual speed and maximal isometric knee extension strength	Study: In men: Time to walk 6metres predicted by WMH volume (beta 0.13, p = 0.02) but not brain atrophy or peak height MTR (adjusted for age and brain size as includes measure of brain atrophy). In women: Usual walking speed predicted by lower MTR height (i.e. indicating abnormal brain tissue) (beta −0.14 (p = 0.01), increased WMH (beta 0.12, p = 0.003) and greater brain atrophy (beta 0.15, p = 0.01) (adjusted for age and brain size). Lower muscle strength associated with peak height MTR (p < 0.005, beta not given).
**30. Aribisala et al. **[61]	2013	UK, LBC 1936 study	694	Longitudinal cohort study	69.5 (0.7) wave 1 and 72.5 (0.7) wave 2	52.9	TBV, ventricular volume, GM, NAWM and WML at wave 2	6 metre walk (normal walking pace) and grip strength at wave 1 and 2	Study: Grip strength at wave 1 significantly predicts ventricular volume at wave 2 (standardized beta −0.10), however there was no significant association with other brain volumes. 6metre walk at wave 1 predicted TBV (−0.07), ventricular volume (0.09), NAWM (−0.07) and WML (0.11) all p < 0.05. Grip strength at wave 2 was associated with ventricular volume (−0.11) and NAWM (0.08). 6 MW at wave 2 was associated with TBV (−0.07), NAWM (−0.09) and WML (0.11) all p < 0.05. Change in physical function between wave 1 and 2 (i.e. decrease in grip strength or increase in 6 MW) was not significantly associated with any brain volume measure. GM volume did not significantly associate with any of the physical function variables at wave 1 or 2. All analyses were adjusted for age, ICV, age 11 IQ, years of education, social class, comorbidity and smoking status. Corrected for false discovery rate.
**31. Rosano et al. **[62]	2011	USA, Cardiovascular health study	643	Longitudinal observational study	72.1-72.6 broken down by BP diagnosis	31-42.7 broken down by BP diagnosis	WMH scale 0-9	Gait speed over 15 ft, starting from standstill. Grip strength of dominant hand.	Study: none, see Rosano (2012), Rosano (2006), Rosano (2005) and Longstreth (1996) for analysis using the Cardiovascular Health study dataset.
**32. Rosano et al. **[63]	2008	USA, Cardiovascular health study	3156	Longitudinal observational study mean f/u 4 years	74 (4.6)	43.2	White matter disease score, brain atrophy score (ventricular enlargement)	Gait speed over 15 ft and grip strength in dominant hand	Study: none, see Rosano (2012), Rosano (2006), Rosano (2005) and Longstreth (1996) for analysis using the Cardiovascular Health study dataset.
**33. Longstreth et al. **[64]	1996	USA, Cardiovascular health study	3658	Longitudinal observational study	70.7 (no sd)	41.7	MR WMSA graded 0-9	Time to walk 15feet, grip strength in dom and non-dom hand	Study: Time to walk 15 ft correlated with white matter grade (0–9) (r = 0.153, p < 0.001), with adjustment for age, sex and presence of clinically silent stroke on MRI. Same model showed no significant associated between grip strength in dom hand or non-dom hand and white matter grade (p > 0.05).

^#^All brain structure variables performed using MRI.

*Associations column key: Study = results published within the study; Given = associations calculated by study authors and supplied to us for this review; Calculated = study authors supplied raw data to us and we performed the analysis.

**Table 4:** Studies identified with brain function and muscle structure

**Author**	**Year**	**Country and dataset**	**n**	**Study design**	**Mean age (sd)**	**Male (%)**	**Brain function**	**Muscle structure**	Associations*
**1. Berryman et al. **[65]	2013	Canada, Training Intervention Study	48	Baseline characteristics from a large physical training intervention study	70.8 (5.4)	41.67	MMSE & modified Stroop test	LBM (DEXA)	Study: none
									Calculated: A GLM showed no association between LBM and MMSE, Stroop naming, reading or inhibition tasks, adjusted for sex and age. However there was an association between the Stroop flexibility task and LBM (t 2.126, p = 0.039, partial eta squared 9.3%), however after adjusting for education and height the effect was attenuated (p > 0.05).
**2. Bites et al. **[66]	2013	Chile	306	Retrospective study	M 74.9 (61–91), F 75.5 (69–90)	24.5	MMSE	TLM, Arm LM and Legs LM (DEXA)	Study: none
									Calculated: Authors sent one data sheet for this study and Bunout et al., as there is a large amount of overlap between the studies. N = 401, mean age 75.3 (sd 4.8), males 28.7%. GLM performed adjusting for sex and gender. Total LM (t 2.38, p = 0.018, partial eta squared 1.4%) and Leg LM (t 3.53, p < 0.001, partial eta squared 3.1%) were both associated with MMSE score but Arm LM is not. After adjusting for height the relationship between total LM and MMSE is non-significant and between leg LM and MMSE is attenuated (t 2.09, p = 0.038, partial eta squared 1.1%).
**3. Bunout et al. **[67]	2005	Chile	298	RCT	M 75.4 (4.8) F 75.8 (4.7)	29.2	MMSE	TLM, Arm LM and Legs LM (DEXA)	Study: none
									Calculated: See Bites et al. 2013 for analysis using this dataset
**4. Auyeung et al. **[68]	2013	Chinese University of Hong Kong - 4y f/u	3153	Prospective observational study	M 71.76 (4.67) F 72.03 (5.07)	49.7	CSI-D and MMSE	ASM, LLMM, FFM (DEXA)	Study: none
									Given: CS-CSID did not predict TLM or ASM at baseline or at 4 years (all p > 0.05). However baseline MMSE was associated with baseline TLM (rho = 0.058, p = 0.002) and ASM (rho = 0.061, p = 0.001) and at follow-up (TLM rho = 0.058, p = 0.002, ASM rho = 0.054, p = 0.005). MMSE at follow up was not associated with TLM or ASM at baseline or follow-up (p > 0.05).
**5. Auyeung et al. **[69]	2011	Chinese University of Hong Kong - 4y f/u	2737	Prospective observational study	M 71.6 (4.58) F 71.5 (4.85)	55.3	CSI-D and MMSE	ASM (DEXA)	Study: In men, low baseline ASM predicted lower MMSE score after 4 years (B = 0.246, p < 0.01) however after adjustment for age, years of education and baseline MMSE it no longer did (p > 0.05). In women, ASM did not significantly predict MMSE after 4 years, either before adjustment or after (p > 0.05).
									Given: see Auyeung et al. (2013) for analysis using this dataset
**6. Lee et al. **[70]	2011	Chinese University of Hong Kong	4000	Prospective observational study	M 72.3 (5.0) F 72.5 (5.3)	50	CSI-D and MMSE	ASM, LLMM, FFM (DEXA)	Study: none
									Given: see Auyeung et al. (2013) for analysis using this dataset
**7. Auyeung et al. **[71]	2008	Chinese University of Hong Kong - baseline	4000	Prospective observational study	M 72.3 (5.0) F 72.5 (5.3)	50	CSI-D and MMSE	ASM (DEXA)	Study: none
									Given: see Auyeung et al. (2013) for analysis using this dataset
**8. Pedersen et al. **[72]	2012	Denmark	72 controls	Cross-sectional study	Median 53 (48–60 inter quartile range)	46	DART, WAIS-III information subtest, TMT-A&B, Rey Auditory Verbal Learning Test (RAVLT), Symbol Digit Modalities Test (SDMT), and fluency tests	FFM (DEXA)	Study: None
									Calculated: FFM did not predict the cognitive z score with or without adjusting for BMI and childhood intelligence (Danish Adult Reading Test, DART). The six individual cognitive tests were then analysed: FFM did not predict RAVLT, SDMT, category fluency (using animals) or TMT-b test, with or without adjusting for BMI and childhood intelligence (DART). Unadjusted, there was no significant association between the letter fluency test (using “s”) and FFM (P > 0.05), however after adjustment for BMI and DART, letter fluency was significantly associated with FFM (t 2.34, p = 0.02, partial eta squared 7.7%). TMT-a test did significantly predict FFM (t 3.08, p = 0.003, partial eta squared 12.3%). After adjusting for BMI and DART the relationship became non-significant.
**9. Magri et al. **[73]	2006	Italy	27 controls	Cross-sectional case–control study	Controls 33.3 (7.15)	0	MMSE	FFM (BIA)	Study: none
									Calculated: FFM did not significantly predict MMSE (p > 0.05), adjusting for age. Adjustments for BMI and educational level did not significantly affect the results.
**10. Lasaite et al. **[74]	2009	Lithuania	29 healthy controls	Observational case–control study	66.2(6.3)	0	TMT-A and B and digit span	FFM (BIA)	Study: none
									Calculated: FFM does not significantly predict TMT-A or B adjusting for age +/− height (p > 0.05). Trend with FFM predicting digit span (t 1.96, p = 0.06, partial eta squared 13%) but attenuated when adjusted for height in addition to age (p = 0.37).
**11. Liu et al. **[75]	2014	Taiwan, I-Lan Longitudinal Aging Study	983	Population based ageing cohort study	65.2 (9.3)	50.6	MMSE	LBM and Relative ASM (=ASM/ height^2^) (DEXA)	Study: A t test comparing mean MMSE in those with normal RASM and those within the lowest 20 % of RASM found a significant difference in men and women of all ages (p < 0.05).
									Given: In a MLR, RASM did not predict MMSE after adjusting for age and sex (beta −0.003, p = 0.940). Adjusting for education in addition did not affect the results.
**12. Moore et al. **[76]	2012	USA, Baltimore Longitudinal Study of Aging	786	Longitudinal cohort study	66.3 (range 26–96)	51.9	California Verbal Learning Test (CVLT), digit-span test, TMT A & B	Mid-femur thigh CSA (CT)	Study: none
									Given: In a linear regression, none of the cognitive tests predicted thigh CSA, adjusting for age and gender. After adjusting for age, gender and height, the digit-span backward test became significantly associated with thigh CSA (beta −1.55, p = 0.024).
**13. Kamijo et al. **[77]	2014	USA, FITKids Study	37 (healthy weight)	Cross-sectional study (case–control substudy comparing obese and healthy weight children)	8.8 (0.6)	46	Kaufman Brief Intelligence Test (K-BIT)	TLM (DEXA)	Study: none
									Calculated: Authors sent one data sheet for the FITKids study as there is considerable overlap in subjects between the two Kamijo et al. papers [77,78], (n = 139, mean age 8.8 (sd 0.6), male 51.1%). A GLM found that TLM did not predict K-BIT after adjustment for age and gender (p > 0.05). Adjusting for BMI in addition did not alter the results.
**14. Kamijo et al. **[78]	2012	USA, FITKids Study	126	Cross-sectional study	8.9 (0.5)	50	Kaufman Brief Intelligence Test (K-BIT)	TLM (DEXA)	Study: none
									Calculated: as per Kamijo et al. [77]

*Associations column key: Study = results published within the study; Given = associations calculated by study authors and supplied to us for this review; Calculated = study authors supplied raw data to us and we performed the analysis.

**Table 5:** Studies identified with measures of brain structure or function and muscle structure or function but no associations given in paper or on request

**Authors**	**Year**	**Country and dataset**	**n**	**Study design**	**Mean age (sd)**	**Male (%)**	**Brain structure or function**	**Muscle structure or function**
***Studies with brain structure and muscle structure (re: *** **Table** 2 ***)***								
**1. Chowdhury et al. **[79]	1994	Sweden	8	Methodology paper	35 (8)	100	Brain volume (CT)	Calculated skeletal muscle volume (CT)
***Studies with brain structure and muscle function (re: *** **Table** 3 ***)***								
**2. Liu-Ambrose et al. **[80]	2010	Canada, Exercise RCT in Vancouver	155	RCT, prospective over 52 weeks	69.6 (2.9)	0	Whole brain volume (MRI)	Gait speed, quads strength and muscle power
**3. Nadkarni et al. **[81]	2012	Canada, Sunnybrook Dementia Study	20 controls	Cross-sectional substudy of longitudinal study	75 (9)	40	Score on Age-Related White Matter Change Scale (MRI)	Self-selected speed on a treadmill
**4. Sullivan et al. **[82]	2005	USA, California, Stanford	51	Case–control study	45.2 (13.9)	100	Caudate, putamen, nucleus accumbens and medial septal / diagonal band volumes and ICV (MRI)	Bilateral grip strength
***Studies with brain function and muscle structure (re: *** **Table** 4 ***)***								
**5. Guthrie et al. **[83]	2004	Australia, The Melbourne Women's Midlife Health Project	1897	9 year prospective, observational population based sample	Median 50	0	Episodic verbal memory using a 10 word recall task (CERAD)	Body composition (DEXA)
**6. Ellis et al. **[84]	2009	Australian Imaging, Biomarkers and Lifestyle (AIBL) study of aging	768 healthy controls	Longitudinal case control study (AD vs MCI vs normal)	70.0 (7.0)	43	CVLT-II, Logical memory, RCFT, digit span, digit symbol coding, D-KEFS verbal fluency, BNT, clock, WTAR, Stroop.	Body composition (DEXA) in subgroup in Perth
**7. Dao et al. **[85]	2013	Canada, Exercise RCT in Vancouver	114	Secondary analysis of RCT data	69.4 (2.9)	0	Stroop test, MMSE	Sub-total lean mass (DEXA)
**8. Schwartz et al. **[86]	2013	Canada, Saguenay Youth Study	983	Longitudinal cohort study	M 14.9 (1.8), F 15.1 (1.9)	48.8	Executive function and Memory	FFM (BIA)
**9. Bagger et al. **[87]	2004	Denmark, PERF study	5607	Prospective, observational cohort study	71.1 (6.6)	0	Short Blessed Test	TLM (DEXA)
**10. Abellan van Kan **[88]	2013	France, EPIDOS study	3025	Prospective multi-centre cohort study	80.51(3.9)	0	SPMSQ	Lean mass and ALM (DEXA)
**11. Nourhashemi et al. **[89]	2002	France, EPIDOS study	7105	Cross-sectional study	80.3 (3.65) (SPMSQ > =8)	0	SPSMQ for orientation, concentration and memory	FFM (DEXA)
**12. Nourhashemi et al. **[90]	2001	France, EPIDOS study	7364	Prospective multicentre study	Broken down by ADLs; means 79.9-82.7 years	0	Pfeiffer’s test (aka SPMSQ)	Body composition (DEXA)
**13. Paolisso et al. **[91]	1997	Italy, Naples	30 (>50y), 30 (75-99y) 19 (>99y)	Observational study	44.5(1.8), 78(0.7), 102(0.8)	46.8	MMSE	FFM (BIA)
**14. Malaguarnera et al. **[92]	2007	Italy, Sicily	66	Placebo controlled, randomized, double-blind, 2-phase study	101(1.3) treatment, 101(1.4) placebo	31.8	MMSE	Total muscle mass (BIA)
**15. Jacobsen et al. **[93]	2012	Netherlands	318	RCT	Mean for each arm given range 73.4-74.0	0	15 words test and Trails B test	BIA and DEXA
**16. Genton et al. **[94]	2011	Switzerland	213 in 1999 and 112 in 2008	Cross-sectional study with 9 year f/u visit	1999 M 71.7(5.2)	1999 49.3	MMSE	FFM (BIA), ASMM (DEXA) and BCM (total body potassium)
					2008 M 80.3(5.2)	2008 49.1		
					1999 F 73.2(5.5)			
					2008 F 82.2(5.6)			
**17. Donaldson et al. **[95]	1996	USA, Baltimore	73	Cross-sectional study	68.8 (7.2)	31.5	MMSE	FFM (DEXA)
**18. Bove et al. **[96]	2013	USA, Boston, Harvard	12	Cross-sectional study	31.6 (6.4)	0	Multiple tests broken down to 5 cognitive domains	Cross sectional area of mid-thigh (CT)
**19. Papadakis et al. **[97]	1995	USA, California, San Francisco	104	Cross-sectional study	75.5(4.9)	100	MMSE, Trails B and DSST	Lean tissue mass (DEXA)
**20. Janssen **[98]	2006	USA, Cardiovascular health study	Baseline 5036	Longitudinal observational study (over 8 years)	65-70 (42.7%), 71–76 (32.7%), 83–89 (18.2%), ≥90 (6.4%)	43.6	MMSE	Whole body muscle mass (BIA) and normalized for height to the skeletal muscle index (SMI, kg/m2)
**21. Masley et al. **[99]	2008	USA, Florida	56	RCT	Controls 43.5 (11.2), Intervention 47.1 (9.4)	Control 39.3, Intervention 53.6	CNS vital signs battery	FFM (BIA)
**22. Houston et al. **[100]	2012	USA, Health, Aging, and Body Composition study	2641	Longitudinal cohort study	74.7 (2.9)	48.9	MMSE	Lean mass (DEXA)
**23. Middleton et al. **[101]	2011	USA, Health, Aging, and Body Composition study	197	Cross-sectional study from a 9 year longitudinal cohort study	Separated into tertile of activity, means range from 73.9-75.8	Not given	3MS	FFM (DEXA)
**24. Koster et al. **[102]	2010	USA, Health, Aging, and Body Composition study	2949	Cross-sectional study from a 9 year longitudinal cohort study	Age 70–79 at baseline	48.5	3MS	Total bone-free lean mass, trunk lean mass, appendicular lean mass (DEXA)
**25. de Rekeneire et al. **[103]	2003	USA, Health, Aging, and Body Composition study	2926	Baseline data from a 9 year longitudinal cohort study	Diabetes mellitis (DM) 73.6 (2.9) and non-DM 73.6 (2.9)	DM 55.9 Non-DM 46.9	MMSE and DSST	Lean mass and lean soft tissue mass (i.e. lean mass minus bone) (DEXA)
**26. de Rekeneire et al. **[104]	2003	USA, Health, Aging, and Body Composition study	Fallers 652, non-fallers 2398	Baseline data from a 9 year longitudinal cohort study	Range 70-79	Fallers 41.7, non-fallers 50.3	Teng Mini-mental State Examination and DSST	Total muscle mass and skeletal muscle mass in the legs (DEXA)
**27. Watts et al. **[105]	2013	USA, Kansas, Brain Aging Project	74 healthy controls	Longitudinal case–control study (Alzheimer’s dementia vs. controls)	74.0 (7.2)	43	MMSE	Lean mass (DEXA)
**28. Canon et al. **[106]	2011	USA, National Health and Nutrition Examination Survey (NHANES)	867	Cross-sectional longitudinal study	Range 60-85	44.8	Digit-symbol coding test	Lean tissue mass (DEXA)
**29. Garry et al. **[107]	2007	USA, New Mexico Aging Process Study	809 rolling participants (average 302 seen per year)	Longitudinal Aging study (1979–2003)	60+ Varied between years	40	3MS (annual), WAIS R digit span, Fuld object memory evaluation, Color Trails 1 and 2, clock drawing (all less than annual)	Annual skeletal tissue mass (DEXA)
**30. Haren et al. **[108]	2008	USA, St Louis, African-American Health Study	124	Population based longitudinal study	56.1(4.4)	100	MMSE, TMT A&B	TLM and ASM (DEXA)
**31. Dvorak et al. **[109]	1998	USA, Vermont	30	Case–control study	73(7)	43.3	MMSE	ASM and FFM (DEXA)

### Association of brain structure and muscle structure

Of the six articles which looked at the relationship between brain structure and muscle structure, three were from the Kansas Brain Aging Project [28-30], and the others were from Germany, UK and USA, Phoenix [26,27,31] (Table 2).

The Kansas Brain Aging Project was set up to determine the effects of exercise and cardiorespiratory fitness on age-related brain changes. Only one of the papers from this project reported the relationship between brain and muscle structure [29]: Burns et al. reported a positive relationship between WBV and TLM (beta 0.20, p < 0.001) when control and subjects with Alzheimer’s disease (AD) were grouped together, adjusting for age sex and intracranial volume (ICV), and they note that this was driven by WM volume [29]. They state that this relationship persists in just the control group but do not give any statistics for this relationship. A General Linear Model (GLM) was performed on the data from the non-demented group supplied to us by the study authors from the Kansas Brain Aging Project [28-30]. WBV, grey matter (GM) volume and hippocampal volume were not predicted by TLM adjusting for age, sex and ICV +/− education. White matter (WM) volume was predicted by TLM (t 3.12, p = 0.003, partial eta squared 14%) adjusting for age, sex and ICV. Adjusting for total years of formal education only slightly attenuated the results (t 2.99, p = 0.004, partial eta squared 13%).

Kilgour et al. also looked at older subjects however they used neck muscle CSA as a measure of muscle bulk [27]. They found that total neck muscle CSA predicted 17% of the variance in whole brain volume (p = 0.01), but they found no significant association between total neck muscle CSA and ventricular, hippocampal or cerebellar volumes (p > 0.05), adjusting for age, sex, ICV and NART (a measure of childhood intelligence).

The other two studies looked at younger subjects. Heymsfield et al. specifically set out to investigate the relationship between brain mass and body composition [26]. They performed multiple linear regression and found that after adjusting for age and fat mass, FFM predicted brain mass in men (beta 0.023, R2 5%, p = 0.01) and women (beta 0.003, R2 6%, p = <0.0001). Fat mass or bone mineral content did not significantly predict brain mass in either sex. So they conclude that it is FFM that drives the relationship between body size and brain size not bone or fat mass. Weise et al. investigated the associations between regional grey matter volume and fat free mass index (FFMI = FFM/height^2^) [31]. They found several areas of grey matter volume that were significantly associated with FFMI (p < 0.01, see Table 2), however after adjusting for percentage body fat or fat mass only two areas remained significant (the right temporal pole and bilateral ventromedial prefrontal cortex).

### Association of brain structure and muscle function

Thirty three studies which included measures of brain structure and muscle function were identified (Table 3). The muscle function variables most commonly studied were grip strength and gait speed. Only one study was identified which used a different measure of muscle function and that was maximal isometric knee extension strength (IKES) [60]. The brain structure variables include: corpus callosum area, and volumes for total and regional GM and WM, cerebrospinal fluid (CSF), cerebellum, hippocampus, basal ganglia and whole brain volume and measures of prevalence of WMH, either volume or scoring systems (e.g. Fazekas).

#### Brain structure and grip strength

The PATH through life project [32-35], the Cardiovascular Health Study [55-57,62-64], the Lothian Birth Cohort 1936 study [61], a study from japan [36] and a study from Philadelphia [37] all looked at the relationship between grip strength and brain structure.

There are four papers identified by our search strategy from the PATH through life project, which was set up to track and define the lifespan course of depression, anxiety, substance use and cognitive ability. In one paper from this project, Anstey et al. (2007) studied the relationship between the area of the corpus callosum (CC) (measured in three sections: anterior, midbody and posterior; and total area) and grip strength [33]. They used the grip strength from the hand the subject wrote with and adjusted for age, sex and ICV. They found no significant relationship between total, anterior or posterior CC area and grip strength however they found a positive relationship between midbody CC area and grip strength (beta −0.09, p < 0.05). They conclude that this is due to the association between midbody CC and the motor cortices. Another paper from the PATH through life project studied the association between grip strength and the percentage of WM occupied by WMH in different brain areas [35]. They found that a larger percentage of WMH per WM volume is associated with decreased grip strength for both the total brain and several brain areas (frontal, temporal, parietal, anterior horn and periventricular body (all p < 0.01)). However, the amount of WMH in the occipital lobe, the cerebellum and the posterior horn was not associated with grip strength. The 2009 paper from this study further investigated the relationship between WMH and grip strength [32]. This time they looked at the relationship in men and women separately. They found that larger amounts of WMH was associated with reduced grip strength, adjusting for age, depression severity and brain atrophy index, in men (p < 0.05) but not in women (n/s). However they comment that they feel that the relationship between WMH volume and motor function is likely to be the same in both sexes and that their finding may be due to the difference in WMH amount between men and women in their study population. Sachdev’s 2006 paper from this study did not look at the relationship between motor function and brain structure and the authors did not respond to our data request [34].

The Cardiovascular Health Study (CHS) is a large, longitudinal, observational study of risk factors for cardiovascular disease in adults 65 years or older, which commenced in 1989 [52]. The CHS measured grip strength and gait speed and WMSA, however only one paper from this study looked at the relationship between grip strength and WMSA [64]. In this paper Longstreth et al. (1996) performed a partial correlation which found no significant association between grade of WMSA (graded on a scale of 0–9) and grip strength in either the dominant or non-dominant hand (p > 0.05) after adjusting for age, sex and presence of clinically silent stroke on MRI [64].

The Lothian Birth Cohort 1936 study measured grip strength at baseline and 3 years later at which point brain volumes were also measured [61]. It is the only study to look at longitudinal changes in muscle strength and brain structure. Grip strength at wave 1 predicted ventricular volume at wave 2 (standardized beta −0.10), however there was no significant association with other brain volumes and grip strength at wave 2 predicted ventricular volume (−0.11) and NAWM (0.08). Therefore, increased grip strength was associated with less brain atrophy in this wave. However, decreased grip strength over 3 years was not significantly associated with any brain volume measure.

The paper by Doi et al. used multiple linear regression to show that grip strength is not related to brain atrophy (beta −0.082 (SE 0.005) p = 0.54) [36]. They measured brain atrophy by mapping the MR brain scans from their subjects to those from healthy controls. Most studies used an index to intracranial volume to calculate degree of brain atrophy. No associations with the other measured brain volumes were included in the paper.

The paper by Hardan et al. looked at the association between caudate volume and grip strength in both hands in children and young adults [37]. They found non-significant statistical trends using Pearson’s correlation between total caudate volume and mean grip strength in the right (r = −0.303, p = 0.05) and left (r = −0.28, p = 0.07) hands. The relationships are negative, therefore there is a trend that those with larger caudate nuclei were found to have lower grip strength in both hands.

#### Gait speed and brain structure

The Sydney Older Person’s Study [38], the TASCOG study [39-41], the Three-City Study [42-45], the AGES-Reykjavik study [60], ABC1921 study [46], WML and mobility study [52,53], further studies from Boston [47-51], the Cardiovascular Health Study [54-57,62-64], the Oregon Brain Aging Study [58,59], the LBC1936 study [61] all looked at the relationship between structural brain measures and gait speed. There were 27 studies identified to include in this section, making it the most researched association in our review. The measurement of gait speed varied considerably, with studies variously using maximum speed or usual pace, and some studies requiring a turn halfway through the measurement and others not. The distance used for the measurement also varied from 2.5 to 75 metres, however the most commonly used measure was usual pace over 6 metres.

The Sydney Older Person’s Study was set up to investigate the environmental, biological and social determinants of healthy ageing. Within it Piguet et al. looked at the relationship between timed walk over 5 meters, adjusted for lower limb arthritis, and cerebellar vermis area (broken down into V1, V2 , V3 and total), and total cerebellar volume. None of the measures of cerebellar size/volume significantly predicted the timed walk [38].

The Tasmanian Study of Cognition and Gait was set up to examine the role of age-related brain changes in causing problems with walking, balance and cognitive abilities in the general community. It measured brain volumes and usual walking speed over 4.6 metres at baseline and 31 months [39]. They found that a greater decline in gait speed over this period was associated with more WM atrophy and hippocampal atrophy and greater accumulation of WML (p < 0.05). There was a non-significant trend with GM atrophy and decline in gait speed (p = 0.06).

The Three-City study is a longitudinal study of the relation between vascular diseases and dementia in persons aged 65 years and older in France, which includes measures of WM volume and maximum walking speed over 6 metres and a repeat walking speed test at the fourth follow up assessment (i.e. roughly 7 years after the first). There were four papers identified from this study which contained reference to these variables.

Soumare et al. looked at the association between WMH volume and both baseline walking speed and decline in walking speed over the 7 year follow up period [45]. They adjusted for age, gender, education and brain white matter volume. They found a significantly lower mean walking speed in those with a total WMH volume above the 75th percentile compare to those below the 25th. They found similar relationships for both deep WMH and periventricular hyperintensities (PVH), however further analyses revealed that PVH may have more of an effect on walking speed than deep WMH. They also looked at WMH volume and the decline in walking speed over the follow up period. They found that having a WMH volume greater than the 90th percentile, more than doubled the risk of decline in walking speed compared with subjects with lower volumes of WMH. This finding was replicated when looking at PVH but not for deep WMH volume. Elbaz et looked at this association further and found that large WMH volumes were not associated with slow walking speed among highly educated participants (OR = 0.72), but were associated with a 2-fold-increased risk of slow walking speed among those with low education (OR = 3.19/1.61 = 1.99) (p interaction = 0.026) [42]. Results remained unchanged after adjustment for height, BMI, and MMSE score.

Dumurgier et al. looked at GM volumes and gait speed in the same cohort and found that only basal ganglia volume (beta 0.075 (SE 0.025) p = 0.003) was significantly associated with walking speed; driven by caudate nucleus volume (beta 0.114 (SE 0.024) p < 0.001) [43]. All other regional GM volumes were not significantly associated with walking speed.

The authors from the Three-City study provided further associations between the variables of interest on written request [39-41]. They looked at the relationship between WM volume and maximal walking speed at baseline, and walking speed decline over 31 months using a multiple linear regression (MLR) and found no significant association. Finally they performed a logistic regression between a one standard deviation increase in WM volume and the risk of having the highest walking speed decline, which was again not significant.

The AGES-Reykjavik study is a longitudinal cohort study which includes an MRI brain and usual walking pace over 6 metres [60]. The MR brain imaging included a magnetization transfer imaging sequence, which can be used to calculate the magnetisation transfer ratio (MTR), which can detect normal and diseased brain tissue by looking at the homogeneity of the brain tissue being studied. They found that in men usual walking speed was predicted by WMH volume (beta 0.13, p = 0.02) but not by degree of brain atrophy or peak MTR height (both p > 0.05) (adjusted for age and brain size) [60]. However in women slower walking speed was associated with: lower MTR height (i.e. indicating abnormal brain tissue) (beta −0.14 (p = 0.01); increased WMH (beta 0.12, p = 0.003); and greater brain atrophy (beta 0.15, p = 0.01) [60]. Additionally they comment that isometric knee extension strength was found to positively correlate with peak height MTR (p < 0.005) however they do not give the strength of the correlation or say what it was adjusted for.

The Aberdeen Birth Cohort 1921 study is a longitudinal study which includes a measure of gait speed (self-paced walk time over 6 metres) and a MR brain scan, which was assessed for WMH. Lower gait speed was significantly associated with increased WMH in the brainstem (p = 0.009, partial eta squared 7%), but not in the cerebral white matter or with PVHs [46].

Seven studies were identified which met the inclusion criteria from the Boston area in the United States. These include two papers from the WML and mobility observational follow up study [52,53], two papers looking at mobility, brain changes and cardiovascular risk factors at baseline [51] and follow up at 2 years [50], two papers conducted at the Beth Israel Deaconess Medical Centre, where it seems there may be overlap between the study volunteers [48,49] and a case–control study about diabetic peripheral neuropathy [47]. The two studies from the WML and mobility study recorded variables at baseline [53] and after a period of follow up (19–22 months) [52]. The baseline paper comments that gait velocity was not significantly predicted by WMSA corrected for ICV, however does not give any specific figure for this analysis [53]. The follow up paper found a significant negative relationship between gait velocity and WMSA at baseline (p < 0.05) [52], however this is in contrast to the baseline paper and only 14 of the original 28 subjects consented for this study. Change in gait speed between visit 1 and 2 did not predict WMSA volume (p = 0.07). They also state they found a significant negative relationship between change in gait speed between visits and CSF volume (r = 0.733, p < 0.005) and a positive relationship between change in gait speed and WM volume (r = 0.558, p < 0.05) [52]. However both the quoted correlations are positive.

Moscufo et al. recruited 99 subjects to a longitudinal study about mobility, brain changes and cardiovascular risk factors [50,51]. Gait speed was measured using time to walk 2.5 metres as part of the Short Physical Performance Battery (SPPB). This is a considerably shorter distance than most other measures of gait speed used. The authors supplied Spearman partial correlations between the brain volumetric variables and gait speed, which were not described in the paper. Greater WMH burden (rho = −0.365, p = 0.0002) and CSF volumes (rho = −0.284, p = 0.004) are associated with slower gait speed. White matter was not found to significantly predict gait speed, however larger GM volume did predict faster gait speed (rho = 0.232, p = 0.020) [51].

An analysis was made in the baseline paper, to investigate whether location of WMH affected gait speed [51]. They selected 10 regions of interest (ROI), which were neural pathways involved in sensory input or motor response and performed a Spearman’s correlation with a corrected significance threshold of ≤0.005 (calculated using the Bonferroni method to adjust for multiple comparisons). All 10 ROI were found to significantly correlate with the walking speed score at p < 0.005 (rho values between 0.279 and 0.426), except in the superior longitudinal fasciculus (p = 0.035) [51].

The follow up paper in this study, performed after 2 years, found that total WMH burden was significantly associated with usual walking speed at baseline but not at follow-up, and maximum walking speed was not associated with total WMH at baseline or follow up [50]. At baseline, regional GM WMH burden in the splenium of corpus callosum and anterior and superior corona radiata, was significantly associated with both usual and maximum walking speed (p < 0.05) and in addition the body of the corpus callosum was also associated with usual walking speed (p < 0.05). At follow-up, WMH burden in the splenium was significantly associated with both walking measures (p < 0.05) and in the body with maximum walking speed. Change in WMH burden, either total or in any of the 7 regional areas, over 2 years was not associated with a decline in usual walking speed (p > 0.1). However decline in walking speed was entered as a binary variable for this analysis (i.e. decline or no decline in walking speed over 2 years), which may have missed a relationship between greater WMH burden and greater declines in walking speed.

Two papers carried out their studies at the Beth Israel Deaconess Medical Centre. One paper looked at healthy volunteers [49] and the other looked at stroke patients in comparison to healthy volunteers [48]. It does not explicitly state the healthy volunteers are the same for each study, but the exclusion criteria, time period and author list would indicate this. The first study measured gait speed over 12 minutes at normal walking speed. MR brain images were analysed for WMH burden and brain volumes corrected for ICV. They found that gait speed was correlated to frontal WM volume (r 0.4, p = 0.003) and frontal grey matter volume (r 0.3, p = 0.01) [49]. However total WMH burden was not associated with gait speed. It is not exactly clear why they looked at frontal brain volumes and gait speed and not other regions of the brain or total brain volume. The second paper also measured gait speed over 12 minutes and used MR brain images. In the non-stroke group, white matter volume was found to predict gait speed (B 1.30, p = 0.03) but not grey matter (p > 0.05). They comment that greater brain atrophy is associated with slower gait speed, but this is for the whole group, so includes stroke patients.

The final study from Boston was by Manor et al. and quoted results from the control group [47]. They found no association between total GM volume or regional GM volumes and walking speed over 75 metres (p > 0.005, Bonferroni adjusted). They were being compared to subjects with diabetic peripheral neuropathy in this study. No results for WM or CSF were reported in the study.

Seven studies from the Cardiovascular Health Study (CHS) met our criteria for inclusion. However three of the studies did not contain any associations between the variables of interest and the study authors did not supply the raw data or correlations [55,62,63]. The first study used gait speed measured over 15 feet, and MR brain images were used to measure ventricular enlargement (VE) and WMH both of which were recorded on a 10 point scale (0–9). Both greater ventricular enlargement (p < 0.001) and greater WMH burden (p = 0.003) were associated with slower baseline gait speed and greater decline in gait speed over the 4 year follow up period [57]. Indeed, after adjusting for baseline performance, those with severe VE were found to have 2.5x the decline in gait speed compared to those with minimal VE at baseline. The model included adjustment for age, sex, race and education.

The next study looked at a subset of the CHS who had undergone two MR brain scans, separated by roughly 5 years, and a MMSE and had undergone assessment on the GaitMat, a 4 metre long instrumented walking surface [56]. THE MR brain scans were classified as above, but given binary cutoffs for the analysis of WMH grade ≥3 or <3 and VE >4 or <4 for some of the analyses. Gait speed was correlated with WMH grade (r = −0.18, p < 0.0001) and with WMH in the brainstem (r = −0.18, p < 0.01). Logistic regression was used to analyse the relationship further and gait speed was separated into quartiles. This showed that those in the lowest two quartiles of gait speed (i.e. < 1.02 m/s) had double the likelihood of having WMH graded 3 or above (p = 0.03). VE graded >4 was not found to be significantly predicted by gait speed, however VE graded > 5 was significantly predicted by gait speed (OR = 2.91 for 1st vs. 4th quartile, OR 3.82 for 2nd vs 4th quartile) [56].

Longstreth et al. is mentioned in the above section on grip strength and brain structure, as this was also studied in this paper [64]. Gait speed was again measured over 15 feet and WMH burden was scored 0–9 on MR brains scans. Time to walk 15 feet was found to correlate with WMH grade (partial correlation coefficient 0.153, p < 0.001, adjusting for age, sex and presence of clinical silent stroke on MR brain) [64]. The population in this study and the study by Rosano et al. [57] overlap considerably and only appear to differ in the time they were still in the study and the particular inclusion and exclusion criteria for that part of the study.

In a separate paper, Rosano et al. found that prefrontal area volume significantly predicted time to walk in a stepwise forward model (beta −0.15, p = 0.02) [54]. This relationship was attenuated when adjustment was made for DSST score, which is a measure of processing speed. They conclude that smaller prefrontal area volume may contribute to slower gait speed through slower information processing.

The final two studies identified are both from the Oregon Brain Aging Study (OBAS) [58,59]. OBAS I is a prospective study commenced in 1989 of healthy older adults age 65 years or older at the initial assessment, a second arm was added in 2004, OBAS II, with less stringent exclusion criteria and these subjects were 85 or older at the start of the study. The first paper by Marquis et al. (2002), looked at the correlation of timed walk, measured at self-selected pace over 9 metres, against brain volumes. Hippocampal volume was found to negatively correlate with timed walk (partial r = −0.12), however no significance value was given and it did not explicitly state what was adjusted for in the correlation [59]. The correlation between TBV and timed walk was <0.1. The other OBAS paper, by Silbert et al. (2008), found that a higher baseline total WMH volume was associated with a greater increase in timed walk over follow up (R^2^ = 0.08, p = 0.0052), the average follow up was 9.1 years [58]. They then looked at whether location of WMH mattered and found that whilst periventricular (PV) WMH volume was associated with a greater change in timed walk over follow up (R^2^ = 0.12, p = 0.0039), a higher subcortical WMH volume was not. These analyses were adjusted for age and ICV. They next looked at change in WMH volume with time and found that a higher rate of accumulation of PV WMH was associated with a greater increase in timed walk (R^2^ = 0.15, p = 0.0453). However there was no relationship described between subcortical or total WMH accrual and change in timed walk. Further data from the study was requested, which was kindly provided. Baseline data from all subjects from OBAS I and II who had had a MRI brain scan and a timed walk at baseline was used to perform our analysis (n = 176).

GLMs were performed to investigate the relationship between brain structures and gait speed, calculated in metres per second for the analysis. In an unadjusted model, gait speed was predicted by TBV, hippocampal volume and WMH volume, all p < 0.001. Upon adjusting for age, sex, ICV and height, TBV (t 3.61, p = 0.004, partial eta squared 4.3%) and WMH (t −2.80, p = 0.006, partial eta squared 4.4%) significantly predicted gait speed, but hippocampal volume did not (p > 0.05).

### Association of brain function and muscle structure

Fifteen papers were identified which looked at brain function and muscle structure: DEXA was used in eleven of the studies; BIA in two; and CT for thigh muscle CSA and MRI for neck muscle CSA in the final two papers (Table 4). Measures of brain function were the MMSE, the Community Screening Instrument of Dementia (CSI-D), Trail Making Test (TMT) A and B, digit span and a measure of global cognitive performance (using z scores from multiple cognitive tests). The studies included the Kansas Brain Aging Project [28-30] and the MHEM study [27], both mentioned in the above section, and studies from Canada [65], Chile [67], the Chinese University of Hong Kong [69,71],Denmark [72], Italy [73], Lithuania [74], Taiwan (the I-Lan Longitudinal Aging Study, ILAS) [75], and from the USA, the Baltimore Longitudinal Study of Aging [76] and the FITKids Study [77,78].

From the Kansas Brain Aging Project, Burns et al. (2010) found a relationship between both MMSE (beta 0.11, p = 0.009) and global cognitive performance (beta 0.12, p = 0.007) and TLM, again grouping AD and control subjects together [29]. They state that in this relationship if the AD subjects are removed from the analysis the results are attenuated, but do not show any results for this. A GLM was performed on the data from the non-demented group supplied to us by the study authors, and we found that neither the global cognitive performance score nor MMSE was predicted by TLM adjusting for age and sex. Adjusting for height and education did not affect this.

The MHEM study used 9 different cognitive tests, which they reduced to two factors using principal components analysis [27]. Total neck muscle CSA did not significantly predict variance in either the memory factor or the cognitive processing factor (p > 0.05), however, it did predict 10% of the variance in the NART score (t = −2.12, p < 0.05). The NART score is a measure of childhood intelligence and the authors comment that the finding is the opposite of what they hypothesized, as they found that lower childhood intelligence is associated with larger neck muscle size in old age.

Berryman et al. supplied the baseline data from their physical training intervention trial. Subjects had performed a MMSE and modified Stroop test and underwent a DEXA scan at baseline [65]. A GLM was performed which showed no association between LBM and MMSE or the Stroop naming, reading or inhibition tasks. However there was an association between the Stroop flexibility task and LBM (t 2.126, p = 0.039, partial eta squared 9.3%), however after adjusting for education and height the effect was attenuated (p > 0.05). This effect was in the opposite direction than might be expected, i.e. bigger muscle mass is associated with a worse score (the Stroop test is measured in seconds to perform the task).

The two papers from Chile are a study by Bunout et al. which includes baseline data for a randomized controlled trial (RCT) investigating an exercise intervention in the elderly [67] and a paper by Bites et al. which used baseline trial data from several studies, including the study by Bunout et al., held on their University database [66]. The authors sent one data sheet for both studies as there is a large amount of overlap between the studies (overlap n = 203). A GLM was performed which showed total LM (t 2.38, p = 0.018, partial eta squared 1.4%) and leg LM (t 3.53, p < 0.001, partial eta squared 3.1%) were both associated with MMSE score but arm LM is not. After adjusting for height the relationship between TLM and MMSE became non-significant and between leg LM and MMSE is attenuated (t 2.09, p = 0.038, partial eta squared 1.1%). Therefore it seems that leg LM is driving the relationship between total LM and MMSE.

Four papers from the Chinese University of Hong Kong were identified which used data from a large prospective longitudinal study looking at bone mineral density in older Chinese adults to assess the relationship between physical and cognitive function [68-71]. They used two measures of cognitive function; the MMSE and the cognitive score from the Community Screening Instrument for Dementia (CS-CSID). Only one of the papers included the associations between the cognitive tests and muscle mass [69]. They found that in men, but not in women, lower appendicular skeletal mass (SM) at baseline predicted lower MMSE at follow up (for a 2.54 kg increase in appendicular SM, there would a 0.246 change improvement in MMSE, p < 0.001). However after adjustment for age, years of education and baseline MMSE, the relationship became non-significant (P > 0.05) [69]. The authors from this study kindly supplied further analyses of their data upon our request.

They performed Spearman’s partial correlations, adjusting for age and sex. There was no significant relationship between baseline CS-CSID and total LM or appendicular LM at baseline or 4 years. However baseline MMSE predicted both baseline total LM (partial rho 0.058, p = 0.002) and appendicular LM (partial rho 0.061, p = 0.001) and 4 years follow up total LM (partial rho 0.058, p = 0.002) and appendicular LM (partial rho 0.054, p = 0.005). However, the effect size is small. They also looked at the whether those with lower MMSE at follow up had lower muscle mass at baseline or follow up but found no significant associations (p > 0.05) [69]. Unfortunately the study authors did not supply data for the relationship between change in cognition and change in muscle mass over the four year follow up which would be very interesting in such a large study population.

Pedersen et al. investigated cognition and physical fitness in normal controls, subjects with impaired glucose tolerance and type 2 diabetes [72]. They supplied the raw data for their control group to be analysed. Subjects underwent DEXA for FFM and six cognitive tests (a cognitive z score was computed as a marker of general cognition). FFM did not predict the cognitive z score with or without adjusting for BMI and childhood intelligence (Danish Adult Reading Test, DART). The six individual cognitive tests were then analysed. There was no association between FFM and most of the individual cognitive tests. Also, unadjusted there was no significant association between the letter fluency test (using “s”) and FFM (P > 0.05), however after adjustment for BMI and DART, letter fluency was significantly associated with FFM (t 2.34, p = 0.02, partial eta squared 7.7%). Letter fluency is a test of executive function and this may indicate that change in this type of cognition with age is associated with FFM in older age. TMT-A test did significantly predict FFM (t 3.08, p = 0.003, partial eta squared 12.3%), but after adjusting for BMI and DART the relationship became non-significant. The TMT-A test is a measure of processing speed.

Magri et al. (2006) performed a cross-sectional study looking at postmenopausal women and HRT, however their study also contained a control group of young healthy women which were used for our analyses after the study authors kindly supplied their data [73]. A GLM was performed with MMSE as the outcome/dependent variable and FFM as the predictor/independent variable as measured by BIA. FFM did not significantly predict MMSE after adjustment for age (p > 0.05) [73]. Adjustments for BMI and educational level did not further affect these results.

Lasaite et al. performed an observational case–control study which looked at women with osteoporosis and healthy controls [74]. The study data for the healthy controls was supplied to us on request. The cognitive measures were the TMT-A and B and a digit span test. FFM was measured using BIA. A GLM was performed on the available data. FFM did not predict TMT-A or B adjusting for age +/− height (p > 0.05). There was a non-significant trend with FFM predicting digit span adjusting for age (t 1.96, p = 0.06, partial eta squared 13%), however when adjusted for height too, the relationship was attenuated (p = 0.37).

The I-Lan Longitudinal Aging Study is an ageing cohort study in Taiwan [75]. Within the study they performed a t test comparing mean MMSE in those with a normal relative appendicular skeletal mass (RASM = ASM/height^2^) with those in the lowest 20% for RASM, and they found a significant difference in both men and women. They also supplied the results of a linear regression on our request for further data, which showed that RASM did not predict MMSE after adjusting for age and sex (beta −0.003, p = 0.940). This may mean there is a non-linear relationship between cognition and muscle mass.

The Baltimore Longitudinal Aging Study is a large longitudinal cohort study, in which the subjects underwent four cognitive tests and had a mid-femur CT for thigh CSA [76]. No associations between the cognitive tests and thigh CSA were included in the study, but the authors sent the results of a MLR they had performed. They found that none of the cognitive tests predicted thigh CSA, adjusting for age and gender. After adjusting for age, gender and height, the digit-span backward test became significantly negatively associated with thigh CSA (beta −1.55, p = 0.024), meaning those with bigger thigh muscles perform better on the test (a higher score is better in the digit span tests).

The final study which looked at cognition and muscle structure is the FITKids study based in Illinois, USA [77,78]. Two papers from this study were identified; however there were no relevant associations in the papers and the study authors kindly provided us with the raw data on which to perform an analysis. As the subjects were all from the same study the authors provided us with one dataset for the study. We performed a GLM which found that TLM did not predict the Kaufman Brief Intelligence Test, used to assess IQ.

## Discussion

This systematic review looked at the evidence for whether: a) brain structure is related to muscle structure, b) brain structure is related to muscle function and c) brain function is related to muscle structure in healthy humans over the life course.

### Brain volumes and muscle mass

The relationship between brain structure and muscle structure was first reviewed (see Table 6 for summary). Three studies tested for an association between whole brain volume and muscle mass; the three papers from the Kansas Brain Aging Project are treated as one study [26-30]. Two studies found a positive association between WBV and muscle mass [26,27] and one study found no significant association [28-30]. However, this study found a significant positive association between WM volume and FFM but no association between GM volume and FFM [28-30]. A different study looked at regional GM volume and found four areas negatively associated with FFM but found most areas to have no association with FFM [31]. Two studies found no association between hippocampal volume and muscle mass [27-30]. One study looked at ventricular volume and cerebellar volume and muscle size and found no association either [27]. Four of the studies were of older adults and two were of younger adults, and it may be that the relationship between brain and muscle structure varies over the life course. Furthermore if there is a relationship between whole brain volume and muscle size it looks like it may be regional brain volume that drives this relationship rather than total volume. The studies are all cross-sectional and a large longitudinal study is needed to explore these relationships further.

**Table 6:** Number of studies of brain structure and muscle structure, direction of effect and number of subjects

	**Negative association *(n)***	**No association *(n)***	**Positive association *(n)***	**References**
**Whole brain size and muscle size**	-	1 *(70)*	2 *(311)*	[26-30]
**White matter volume and muscle size**	-	-	1 *(70)*	[28-31]
**Grey matter volume and muscle size**	1 (76)*	2 (146)*	-	[28-31]
**Hippocampal volume and muscle size**	-	2 *(121)*	-	[27-30]
**Cerebellar volume and muscle size**	-	1 *(51)*	-	[27]
**Ventricular volume and muscle size**	-	1 *(51)*	-	[27]

*This study found negative associations between some areas of grey matter volume (the right temporal pole and bilateral vmPFC) and muscle size but found the majority of GM areas had no association.

### Brain structure and muscle function

Next evidence for an association between muscle function and brain structure was reviewed. Muscle function was either grip strength (5 studies, see Table 7 for summary) or gait speed (13 studies, see Table 8 for summary) apart from in one paper where isometric knee extensor strength (IKES) was used [60].

**Table 7:** Number of studies of brain structure and grip strength, direction of effect and number of subjects

	**Negative association *(n)***	**No association *(n)***	**Positive association *(n)***	**References**
**Grip strength and whole brain volume**	-	1 *(694)*	-	[61]
**Grip strength and WM volume**	-	1 *(694)*^*a*^	1 *(694)*^*a*^	[61]
**Grip strength and GM volume**	-	1 *(694)*	-	[61]
**Grip strength and caudate volume**	-	1 *(41)*	-	[37]
**Grip strength and ventricular volume/brain atrophy**	2 *(804)*	-	-	[36,61]
**Grip strength and WMH**	1 *(478)*	2 *(4352)*	-	[35,61,64]
**Change in grip strength over f/u and whole brain volume**	-	1 *(694)*	-	[61]
**Change in grip strength over f/u and WM volume**	-	1 *(694)*	-	[61]
**Change in grip strength over f/u and GM volume**	-	1 *(694)*	-	[61]
**Change in grip strength over f/u and ventricular volume**	-	1 *(694)*	-	[61]
**Change in grip strength over f/u and WMH volume**	-	1 *(694)*	-	[61]

^a^At wave 1 in this study there was no association and at wave 2 there was a positive association [61].

**Table 8:** Number of studies of brain structure and gait speed, direction of effect and number of subjects

	**Negative association *(n)***	**No association *(n)***	**Positive association *(n)***	**References**
**Gait speed and whole brain volume**	-	-	2 *(885)*	[58,59,61]
**Gait speed and WM volume**	-	2 *(1587)*	3 *(813)*^b^	[42,48-50,61]
**Gait speed and GM volume**	-	4 *(2449)*^c^	3 *(367)*^b,d^	[43,47-50,54,61]
**Gait speed and hippocampal volume**	-	1 *(191)*	-	[58,59]
**Gait speed and cerebellar volume**	-	1 *(111)*	-	[38]
**Gait speed and WMH volume**	7 *(7145)*^e^	4 *(278)*^e,f^	-	[45,46,49,50,52,53,56-61,64]
**Gait speed and CSF volume/ventricular volume/brain atrophy**	3 *(3221)*^g^	2 *(1489)*^g^	-	[50,56,57,60,61]
**Gait speed and WMH progression over f/u**	-	1 *(14)*	-	[52]
**Change in gait speed over f/u and whole brain volume**	-	1 *(694)*	-	[61]
**Change in gait speed over f/u and WM volume**	-	2 *(1622)*	-	[42,61]
**Change in gait speed over f/u and GM volume**	-	1 *(694)*	-	[61]
**Change in gait speed over f/u and CSF/ventricular volume**	-	1 *(694)*	1 *(2450)*	[57,61]
**Change in gait speed over f/u and WMH volume**	-	1 *(694)*	2 *(4152)*	[45,57,61]
**Change in gait speed over f/u and WM atrophy**	-	-	1 *(225)*	[39]
**Change in gait speed over f/u and GM atrophy**	-	1 *(225)*	-	[39]
**Change in gait speed over f/u and hippocampal atrophy**	-	-	1 *(225)*	[39]
**Change in gait speed over f/u and WMH progression**	-	1 *(77)*	1 *(225)*	[39,50]

^b^one study only looked at frontal WM/GM volume not total WM volume [49].

^c^basal ganglia volume was positively associated with gait speed in this study [43].

^d^one study only looked at prefrontal area volume within GM [54].

^e^this study found usual walking speed was negatively associated with WMH but that maximum walking speed was not [50].

^f^except brainstem WMH which were negatively associated with gait speed in this study [46].

^g^at wave 1 there was a negative association and at wave 2 there was no association in this study [61].

#### Brain structure and grip strength

Only one study looked at the relationship between whole brain, GM or WM volume and grip strength [61]. There were no significant associations except for a positive relationship between WM volume and grip strength at wave 2 (age 73) [61]. This could mean that the relationship between WM volume and grip strength only becomes important with age, once a volumetric threshold is passed. Another study found no association between caudate volume and grip strength [37]. However the basal ganglia may be expected to play less of a role in grip strength than in gait speed. Two studies found a negative association with markers of brain atrophy and grip strength [36,61], however one of these studies also looked at change in grip strength over 3 years and found no association with ventricular volume (a marker of brain atrophy) [61]. This means that whilst cerebral atrophy and grip strength appear to be associated, decline in grip strength does not predict cerebral atrophy. A longitudinal study including both measures would help explain this relationship further.

One study found an association between WMH and grip strength [35]. They found that location of the WMH is important, with some brain areas correlating with grip strength and others not [35]. On looking at the data separated by sex, this relationship persisted in men, but not women, but the study authors think this is due to a sex difference present in their study population, with the men having higher volumes of WMH [32]. Two larger studies found no association between WMH and grip strength, however one of these studies used a visual rating scale from 0–9 to measure WMH, which may lead to differing results than using WMH volumes [61,64]. The other study also looked at change in grip strength over 3 years and WMH volume at follow up and found no association [61]. WMH are known to predict dementia and cerebrovascular disease but their relationship to physical function is less well understood [110].

#### Brain structure and gait speed

Two studies found a positive association between WBV and gait speed [58,59,61], whereas studies investigating the relationship between WM and GM volume and gait speed found less unanimous results. Three studies found a positive association between WM volume and gait speed [48,49,61] and two studies found no association [42,50]. Four studies found no association between GM volume and gait speed [43,47,48,61] but three studies found a positive relationship [49,50,54]. There was no evidence that hippocampal volume or cerebellar volume were associated with gait speed [38,58,59]. It may be that specific sub-regions of the white and grey matter are associated with gait speed, for example one paper found an association between basal ganglia volume and gait speed but no association with total GM and gait speed. Further studies looking at regional brain areas will help to clarify these relationships. Five studies looked at markers of brain atrophy and gait speed; two found a negative association (i.e. more atrophy associated with a slower gait speed) [50,56,57] and one found no association [60], with one finding an association at wave 1 but not at wave 2 [61].

No association was found between change in gait speed over follow up and whole brain, WM and GM volume (mean length of follow up in each study, 3 and 7 years) [42,61]. However one large study did find an association between ventricular volume and change in gait speed over follow up (mean 4 years) [57] but another study found no association (mean follow up 3 years) [61]. Only one study looked at the relationship between change in gait speed and change in brain structure over time (mean follow up 30.6 months) [39]. They found a positive association between change in gait speed and WM and hippocampal atrophy but no association with GM atrophy. The well-established relationship between cognitive decline and gait speed and cognitive decline and brain atrophy could underpin the possible relationship between brain atrophy and gait speed [4-8]. It is interesting that the only study to look at both variables in a longitudinal study found significant associations between brain structure and gait speed and further studies like this are needed.

Eleven studies were found which looked at gait speed and WMH, making it the most studied relationship in our review. Seven of these studies found that greater levels of WMH were associate with slower gait speed [45,50,52,56-61,64], but four other smaller studies found no association [46,49,50,53]. Two of these studies found that this is primarily due to the volume of PVH and not subcortical WMH lesions [45,58] and two papers found that volume of brainstem WMH was associated with gait speed [46,56]. One small study (n = 14) found no association between gait speed and WMH progression over follow up (19–22 months) [52]. However, change in gait speed was found to be associated with WMH volume in two large studies [45,57] with another study showing no association [61]. Two studies looked at change in both variables; one found that greater decline in gait speed was associated with greater WMH progression [39], whereas the other found no association [50]. Further studies looking not just at total WMH volume but their rate of accumulation and location within the brain, and their association with gait speed should help clarify this area.

### Cognitive function and muscle mass

Nine studies were found which looked at cognitive function and muscle structure. Table 9 shows the main results from these studies. Three studies looked at a measure of global cognitive performance (a composite score of several tests used in their study) and muscle size and all 3 found no association [27,28,72]. The Kaufman Brief Intelligence Test can also be used as a marker of general cognition and it too found no association with muscle size [77,78]. Seven studies looked at muscle size and MMSE score, which is a useful screening tool for dementia but is not a robust test of cognitive function. Four of the studies found no association between MMSE and muscle mass [28,65,73,75] and one found an association but with a very small effect size [68]. However in one study which showed no association between MMSE and FFM, when comparing subjects with normal RASM and those within the lowest 20% of RASM this study found a significant difference in mean MMSE [75]. Several of the included studies did not include those with cognitive impairment and it may be that an association does exist between MMSE and muscle size but in a non-linear relation, affecting the frailer older adult more, but that it was not picked up in these studies due to the method of analysis in a linear regression. Overall though in healthy individuals it seems that no such association exists. The final study found an association between leg LM and MMSE but not between total or arm LM [66]. Sarcopenia is known to affect leg and arm muscles differently which perhaps explains this effect [111]. Another screening tool for dementia, the cogscore part of the CSI-D, also found no association with muscle mass [68]. It is well established that gait speed and cognition are associated in older age and these results appear to show that muscle size is not a driving force behind this relationship [4-6].

**Table 9:** Number of studies of cognition and muscle size, direction of effect and number of subjects

	**Negative association *(n)***	**No association *(n)***	**Positive association *(n)***	**References**
**Muscle size and global cognitive score**	-	3 (193)	-	[27,28,72]
**Muscle size and MMSE**	-	5 (1434)^h,i^	2 (3459)^h^	[28,65,66,68,73,75]
**Muscle size and California Verbal Learning Test**	-	1 (786)	-	[76]
**Muscle size and CSI-D**	-	1 (3153)	-	[68]
**Muscle size and digit span**	-	2 (815)^j^	-	[74,76]
**Muscle size and Kaufman Brief Intelligence Test**	-	1 (139)	-	[77,78]
**Muscle size and modified Stroop test**	-	1 (48)	-	[65]
**Muscle size and NART**	1 (51)	-	-	[27]
**Muscle size and TMT-a**	-	3 (887)	-	[72,74,76]
**Muscle size and TMT-b**	-	3 (887)	-	[72,74,76]

^h^Leg LM was associated with muscle size but not total LM or arm LM in this study [66].

^i^In an MLR there was no association between MMSE and FFM but when comparing subjects with normal RASM and those within the lowest 20% of RASM this study found a significant difference [75].

^j^Digit span forwards unadjusted and adjusted was not associated with thigh muscle CSA, but adjusted backwards digit span was negatively associated with thigh muscle CSA in this study [76].

With regard to the individual cognitive tests (which measure processing speed and executive function), there were no significant associations [65,72,74,76], except for the NART (a measure of childhood IQ, which showed a negative association with neck muscle CSA [27]. The authors comment that perhaps subjects with higher cognition are more likely to have sedentary jobs and therefore more likely to lose their muscle mass over time. None of the studies looking at cognition and muscle size contained longitudinal associations therefore whilst these results appear to support no association between muscle mass and cognition; it may be that longitudinal data would show an association, whereby those that lose more muscle with age have a sharper slope of decline in their cognition also. Longitudinal studies will help to elucidate these complex relationships further.

### Limitations

In the review protocol the decision was made to write to study authors for relevant associations or data that were not given in the study but could be calculated using the recorded variables. This expanded the number of articles included in the review and the scope that they covered, however this may have led to some bias in which articles responded to the request and therefore what was reported, as study authors who found an association may have been more likely to reply. Of the 79 articles written to 59 replied therefore 25% did not respond. However the studies that did respond included both those which showed a significant association and those that did not. The associations which were sent to us and the associations performed by us have not undergone peer review (e.g. for variable selection when adjusting the models), however we have included this information in our review and the statistical technique used to remain as transparent as possible.

The studies included in the review used a wide variety of techniques to record the variables of interest which means it is difficult to compare them (e.g. in a meta-analysis). Gait speed for example was recorded over multiple lengths, using automated and manual techniques and different levels of speed (i.e. maximum or usual pace). The large differences in how gait speed was measured combined with the fact that over longer distances it can become a test of cardiovascular fitness etc. more than a test of muscle function, makes it difficult to compare the results of these studies directly. Hopefully more standardized testing will come about in the wake of resources like the NIH toolbox which includes a proforma for measuring gait speed [112].

When looking at the relationship between brain size and muscle size or function it is important to make sure that the size of the individual being studied is not acting as a confounding factor (i.e. that large people have large brains and large muscles). This meant that it was important to ensure that some measure of body size had been adjusted for in each association (e.g. ICV, height, BMI). In most of the studies we looked at this occurred but in some it did not and this may lead to a false relationship being reported.

A relatively wide range of ethnicities are represented in the study, however Caucasian subjects were by far the most commonly studied and there were no studies including those of Arabic or Indian ethnicity. Also most of the studies used subjects in their sixties, seventies or eighties, meaning the validity of our findings for other age groups, particularly children and adolescents is limited.

Finally, while a few of the studies included longitudinal data, it would be very useful to have more studies looking at the relationships over time as these may be able to highlight potential modifiable factors.

## Conclusions

An increasing body of research has now linked brain function (cognition) and muscle function (e.g. gait speed) [4-8], however less well studied is the role of muscle and brain structure in this relationship. This systematic review looks at the evidence for whether: brain structure is related to muscle structure; brain structure is related to muscle function; and brain function is related to muscle structure in healthy humans across the lifecourse.

The review found evidence of a positive association between whole brain volume and total white matter volume with muscle size and evidence that some areas of regional grey matter volume (right temporal pole and bilateral vmPFC) are negatively associated with muscle size [26-31].

The review found no evidence of a relationship between grip strength and whole brain volume, however there was some evidence of a positive association between grip strength and WM volume. Markers of brain ageing, that is brain atrophy and greater WMH accumulation, were associated with grip strength [35,36,61]. Unlike grip strength, there is evidence that gait speed is positively associated with whole brain volume; this relationship may be driven by total WM volume or regional GM volumes, specifically the hippocampus [58,59,61]. Like grip strength, gait speed is also associated with markers of brain aging; WMH accumulation, brain atrophy and WM atrophy all show evidence of either a temporal association with gait speed or change in gait speed with time, with PVH and brainstem WMHs playing a particularly important role, but not subcortical WMH [45,57].

The evidence overwhelmingly points to no association between cognition and muscle size, except in the case of MMSE where it is mixed, but MMSE is more a screening tool for dementia than a true marker of cognitive function [27,28,65,66,68,72,73,75]. Longitudinal studies are now needed to explore these relationships over time, which will allow a better understanding of the potential causal relationships.

## Appendix 1 Medline Search

1. brain/ or exp brain stem/ or exp cerebral ventricles/ or exp limbic system/ or exp mesencephalon/ or exp prosencephalon/ or exp rhombencephalon/

2. (brain adj3 volume).tw.

3. white matter.tw.

4. mental processes/ or cognition/ or cognitive reserve/ or comprehension/ or executive function/ or higher nervous activity/ or maze learning/ or exp memory/ or thinking/ or decision making/ or judgment/ or problem solving/

5. Intelligence/

6. exp aptitude tests/ or exp neuropsychological tests/

7. cognitive function.tw.

8. muscles/ or muscle, skeletal/ or abdominal muscles/ or rectus abdominis/ or deltoid muscle/ or neck muscles/ or pectoralis muscles/ or psoas muscles/ or quadriceps muscle/ or rotator cuff/

9. Body Composition/

10. muscle cross sectional area.tw.

11. exp Muscular Atrophy/

12. exp Muscle Strength/

13. exp Walking/

14. muscle power.tw.

15. Physical Fitness/

16. physical performance.tw.

17. 1 or 2 or 3

18. 4 or 5 or 6 or 7

19. 8 or 9 or 10 or 11

20. 12 or 13 or 14 or 15 or 16

21. 17 and 19

22. 17 and 20

23. 18 and 19

24. 21 or 22 or 23

25. limit 24 to humans

26. limit 25 to case reports

27. 25 not 26

## Abbreviations

6 MW: Six metre walk test; AD: Alzheimer’s disease; ALM: Appendicular lean mass; ASM: Appendicular skeletal mass; BIA: Bioimpedance analysis; BMI: Body mass index; CC: Corpus callosum; CSF: Cerebrospinal fluid; CSI-D: Community screening instrument of dementia; CVD: Cardiovascular disease; FFM: Fat free mass; GLM: General Linear Model; GM: Grey matter; ICV: Intracranial volume; IKES: Isometric knee extension strength; K-BIT: Kaufman Brief Intelligence Test; LM: Lean mass; LLMM: Lower limb muscle mass; MLR: Multiple linear regression; MMSE: Mini mental state examination; MTR: Magnetisation transfer ratio; NAWM: Normal appearing white matter; PVH: Periventricular hyperintensities; RASM: Relative appendicular skeletal mass; RCT: Randomized controlled trial; ROI: Region(s) of interest; SM: Skeletal mass; SPPB: Short physical performance battery; TBV: Total brain volume; TLM: Total lean mass; TMT: Trail making test; VE: Ventricular enlargement; WBV: Whole brain volume; WM: White matter; WMH: White matter hyperintensities; WML: White matter lesions; WMSA: White matter signal abnormalities.

## Competing interests

The authors declare that they have no competing interests.

## Authors’ contributions

AHMK and JMS proposed the hypotheses and drafted the manuscript. AHMK and OMT performed the longlisting and shortlisting of the included studies. AHMK wrote the data extraction sheet, compiled the tables and performed the data extraction and data analysis. All authors approved the final manuscript.

## Pre-publication history

The pre-publication history for this paper can be accessed here:

http://www.biomedcentral.com/1471-2318/14/85/prepub
